# Acknowledgement to Reviewers of *Genes* in 2016

**DOI:** 10.3390/genes8010030

**Published:** 2017-01-13

**Authors:** Genes Editorial Office

**Affiliations:** MDPI AG, St. Alban-Anlage 66, 4052 Basel, Switzerland; genes@mdpi.com

The editors of *Genes* would like to express their sincere gratitude to the following reviewers for assessing manuscripts in 2016.

We greatly appreciate the contribution of expert reviewers, which is crucial to the journal’s editorial process. We aim to recognize reviewer contributions through several mechanisms, of which the annual publication of reviewer names is one. Reviewers receive a voucher entitling them to a discount on their next MDPI publication and can download a certificate of recognition directly from our submission system. Additionally, reviewers can sign up to the service Publons (https://publons.com) to receive recognition. Of course, in these initiatives we are careful not to compromise reviewer confidentiality. Many reviewers see their work as a voluntary and often unseen part of their role as researchers. We are grateful to the time reviewers donate to our journals and the contribution they make.

If you are interested in becoming a reviewer for *Genes*, see the link at the bottom of the webpage http://www.mdpi.com/reviewers.

The following reviewed for *Genes* in 2016:
Abdel-Wahab, OmarLiu, QingAbel, Jean M.Liu, YuanAchermann, PeterLiu, YuatoAgarwal, SaurabhLoesch, Danuta Z.Ahn, Eun-Young ErinLöffler, MarkusAkiyama, MasahiroLoizou, Joanna I.Alarcon, VernadethLovett, SusanAlevizos, IliasLozano, ReymundoAlexeyev, Mikhail F.Lozano-Velasco, EstefaníaAllan, JamesLu, Lin-YuAllers, ThorstenLuco, Reini F.Allred, Nicholette DanielleLucretti, SergioAlmouzni, GenevieveLudwig-Müller, JuttaAlonso, Laura CristinaLuján, HugoAlves, Carla SophiaLunter, GertonAmodeo, AmandaLuo, XuelianAn, Shi-qiMaas, AngelaAndreassen, Paul R.Machado, Artur Da CâmaraAoki, KohMack, StevenApichai, TuanyokMadireddy, AdvaithaAqeilan, Rami I.Madsen, OleAraujo, Sofia J.Maga, GiovanniArs, ElisabetMahimainathan, LeninAustin, CarolineMajid Hafezparast, MajidAutexier, ChantalMakarevitch, IrinaAwadalla, Mona S.Malik, RainerAy, FerhatManakil, JaneAylon, YaelMangolini, AlessandraAzzalin, ClausMarci, RobertoBaba, HideoMaréchal, AlexandreBagchi, AnindyaMariadason, John M.Baharoglu, ZeynepMarondedze, ClaudiusBakay, MarinaMartinat, CécileBandini, ErikaMartínez, PaulaBanerjee, TaraswiMartinussen, IngerBangera, RamaMasepohl, BerndBanilas, GeorgiosMassie, CharlieBao, ManzhuMasuda, KiyoshiBarac, AnaMasutomi, KenkichiBarbon, AlessandroMazel, DiddierBarbosa, EudesMcCallum, SusanBarnett, TimothyMcCollum, DannelBassil, Nahla V.McCown, Thomas J.Batchu, Ramesh B.McGrann, GrahamBates, DavidMcintosh, RobertBatsché, EricMcMahon, FrancisBauer, JohannMcVey, MitchBeal, PeterMehlig, KirstenBelamkar, VikasMellon, IsabelBello, DhimiterMelone, Mariarosa A. B.Ben-Dov, Iddo Z.Mendez, JuanBenfield, David A.Merino, GraciaBerger, WalterMerrikh, HouraBergthaler, AndreasMey, Alexandra R.Berkhout, BenMeyre, DavidBermejo, RodrigoMica, EricaBetts, DeanMichel, BénédicteBeversdorf, DavidMichelhaugh, Sharon K.Bi, XinMilà, MontserratBierzynska, AgnieszkaMilek, MihaBishop, DouglasMillard, AndrewBissonnette, NathalieMiller, DennisBlanco, LuisMiller, Douglas C.Blandino, GiovanniMiotto, BenoitBochman, Matthew L.Mitchell, Braxton D.Borodovsky, MarkMiyadera, KeikoBottema, CynthiaMiyamoto, ToshinobuBou-Gharios, GeorgeMizuo, KeisukeBouhassira, EricMody, VickyBousman, ChadMonteys, Alejandro MasBouveret, RomaricMoriguchi, TakayaBowser, Robert P.Morona, RenatoBoye, ErikMorretti-Ferreira, DaniloBoye, Sanford L.Movassat, JamilehBraconi, ChiaraMulle, Jennifer G.Brannigan, Robert EMurphy, TomBrosh, Robert M.Murray, HeathBrown, GrantNagaoka, SumiharuBrown, SusanNagata, ShinjiBuckley, PeterNagy, IstvanBundschuh, RalfNakamura, MasatakaCaccamo, DanielaNakamura, ToruCahoon, Aubrey BruceNaldini, AntonellaCalcutt, MichaelNana-Sinkam, PatrickCalviello, GabriellaNanba, EijiCalzone, LaurenceNandi, SaikatCanevari, SilvanaNapierala, MarekCannon, Steven BNargang, FrankCaramalho, IrisNazar, Ross N.Cardenas, AndresNelissen, HildeCarr, AntonyNelson, Christopher P.Carraro, NicolaNepal, MadhavCarulli, L.Nilsson, PeterCastaldi, AlessandraNishiguchi, MasamichiCastandet, BenoitNitiss, JohnCastiglioni, IsabellaNoble, Janelle A.Castrén, Maija L.Nolin, Sarah L.Cavallini, GiorgioNoordam, RaymondCazier, Jean-BaptisteNoyes, Richard D.Cerri, MarionNúñez Garrote, José IgnacioChallagundla, Kishore B.O’Connor, MatthewChan, AgnesO’Donnell, MichaelChan, DylanO’Keefe, Joan A.Chan, Raquel L.O’Rourke, Jamie A.Chang, Nan-ShanOnengut-Gumuscu, SunaChapman, Mark AOsbourn, AnneChen, Yen-HaoOxnard, GeoffreyCheng, DePabinger, StephanCheng, LiangPacak, AndrzejChilosi, MarcoPadovan, AmandaChristelová, PavlaPapantonis, ArgyrisChung, EuiryongPardini, BarbaraCiaudo, ConstancePark, ChankyuCommichau, Fabian M.Pasero, PhilippeCondon, CiaránPastore, LisaCong, YushengPattekari, PravinConklin, DanielPatterson, StevenConn, JanPavlov, YouriCordenonsi, MichelangeloPedergnana, VincentCouser, NatarioPeffers, Mandy J.Crosby, HeidiPelczar, PawelCrouch, RobertPellegrini, LucaDallinger, ReinhardPellicioli, AchilleDane, FennyPeltonen, SirkkuDar, Altaf A.Pemberton, TrevorDavidson, Nicholas O.Peprah, EmmanuelDavies, KayPerálvarez Marín, AlexDavis, Michael E.Perez Pascual, DavidDavoli, RobertaPérez-Pascual, DavidDavydova, JuliaPerochon, AlexandreDawid, SuzannePerumal, DeepakDe Bortoli, MichelePfander, BorisDe Bruin, Robertus A.M.Pickett, Hilda A.De Gennaro, LuigiPiñero-Dalmau, DanielDe Piccoli, GiacomoPlohl, MiroslavDe Rossi, AnitaPociot, FlemmingDe, SubhajyotiPodar, KlausDeclercq, JeroenPopovich, DavidDennis, JonPortman, MichaelDias, RicardoPourquier, PhilippeDiener, Kerrilyn R.Prasanth, SupriyaDing, LinPratas, DiogoDinh, ThanhPricl, SabrinaDobrovic, AlexanderProsseda, GianniDodds, Ken G.Rafael-Fortney, Jill A.Doering, LaurieRakoczy, Elizabeth P.Dombrowski, James E.Ramachandran, SrinivasanDonaldson, Anne D.Ramsahoye, BernardDong, JixinRapacz, MarcinDonnelly, ChristopherRausch, Jason W.Donnelly, DeirdreRechkoblit, OlgaD’Onofrio, GiuseppeReddel, RogerDoyle, SiamsaReeves, MatthewDu, XiaojunRennert, OwenDundas, IanRhind, NicholasDunner, S.Rivero, RosaDuprez, DelphineRoca, XavierEamens, AndyRodriguez-Revenga, LaiaEberl, LeoRogers, Lesley J.Ebrahimie, EsmaeilRomagnani, PaolaEdwards, MartinRose, Laura E.Ehmer, UrsulaRosenkranz, DavidEijkelkamp, BartRouhi, ArefehEl-Hage, NaziraRoy, SouravEl-Heliebi, AminRoy-Chaudhuri, BiswajoyEllis, LeighRubio, FranciscoEroglu, ZeynepRubis, BlazejEstevez, AntonioRudolph, ChristianEwen, JoshuaRuf, SabineFaenza, IreneRuprich-Robert, GwenaëlFaghihi, MohammadRussell, PaulFaherty, Christina SuzanneRusso, AntonellaFanini, FrancescaRuwe, HannesFarina, NicholasRyan, JoanneFederle, MichaelSabina, HammerFeil, RobertSachidanandam, RaviFeng, HalianSaez, EnriqueFerracin, ManuelaSaffran, Wilma A.Figueroa-Romero, ClaudiaSaha, SuryaFitches, ElaineSahle, SvenFitzpatrick, Elizabeth A.Saitoh, ShinjiFleming, Leah R.Sale, JulianFlisikowska, TatianaSalmon-Divon, MaliFolco, Hernan DiegoSalvatore, FrancescoFoletta, Victoria C.Sampedro Jiménez, JavierForoud, NoraSamuel, Charles E.Foster, TimothySamuels, IvyFozo, ElizabethSantamaría, EstrellaFranco, LuisSaretzki, GabrieleFranzè, AnnamariaSasaki, HirokiFraser, Malcolm J.Satoh, ShigeruFredriksson, RobertSchalinskie, KevinFreudenreich, Catherine H.Schalk, Isabelle J.Frugier, FlorianSchallenberg-Rüdinger, MareikeFujita, MasatoshiSchultze, Joachim L.Fusco, AlfredoSchwarz, Erika N.Gaken, JoopSchweickert, AxelGalindo, ReneSclavi, BiancaGallitano-Mendel, AmeliaSearle, JeremyGambino, G.Sebastiani, FedericoGargus, John JaySegall, Jeffrey E.Gauthier, BenoitSha, ZheGeorgakilas, Alexandros G.Shalaby, ShahinazGerdol, MarcoSharakhov, IgorGibbons, RichardSharkey, Thomas D.Gifford, Alex H.Shavrukov, YuriGilbert, Sarah C.She, QunxinGilley, David P.Shea, PatrickGilson, EricShelest, EkaterinaGinn, Samantha L.Shirasawa, KentaGiri, Veda N.Shiratori, HidetakaGiuliani, AlessandroShuman, HowardGoldammer, TomShy, Michael E.Goldmann, WilfredSieber, JessicaGoldstein, RonaldSieving, Paul AlbertGomes Rocha, AgostinhoSilar, PhilippeGomez, DavidSilliker, MargaretGonzález-Tizón, Ana M.Skibbens, RobertGott, Jonatha M.Skot, LeifGowik, UdoSmagghe, GuyGoya, Gerardo F.Smith, DarrenGraham, DavidSmith, DavidGraves, DavidSmith, EdvardGray, Phillip N.Smith, Joshua A.Greb, ThomasSmits, Wiep KlaasGreiner, StephanSnowden, JessicaGriesenbach, UtaSoares, PaulaGrigorenko, ElenaSobol, Robert W.Grogan, DennisSoitamo, Arto J.Grzebelus, DariuszSojka, DanielGu, WenyiSokolowski, MarcusGünes, CagataySolt, Laura A.Gupta, RisheinSong, GuoqingHaendeler, Jojo (Judith)Souček, PavelHagen, Stephen J.Spies, MariaHagerman, RandiSpyridopoulos, IoakimHalazonetis, Thanos D.Srihari, SriganeshHammani, KamelStanta, GiorgioHampson, David R.Stellos, KonstantinosHancock, Dana B.Stieger, KnutHande, M. PrakashStoneking, MarkHannon, EilisStracker, Travis H.Hansen, ChristianStracy, MathewHardies, Stephen C.Strano, SabrinaHaro, DiegoStuart, JeffHarrington, CharlesSturgill, David M.Hart, David A.Sudol, MariusHarushima, YoshiakiSugimura, HaruhikoHasegawa, Paul MichaelSuh, Dae-yeonHatzopoulos, PolydefkisSung, Huang-MoHazen, Tracy H.Sung, PatrickHe, ShanSurtees, Jennifer A.Heaphy, ChristopherSwevers, LucHeber, DavidSymington, LorraineHegde, Muralidhar L.Takahashi, TakuHergovich, AlexanderTakahashi, TatsuroHernández-Alcoceba, RubénTam, PatrickHesson, LukeTang, RenjieHirakawa, HidekiTang, YuHiroi, NoboruTassone, FloraHishida, TakashiTassone, PierfrancescoHitchins, MeganTaylor, BrandieHleap, Jose SergioTaylor, ElaineHodge, SusanTaylor, Shirley M.Hoeper, DirkTelias, MichaelHoffmann, KlausTergaonkar, VinayHolloman, WilliamThompson, BarryHolvoet, PaulThompson, LukeHorikoshi, MomokoThorpe, ChavaunneHorn, Rebekah L.Tien, Hwei-FangHorvath, David P.Titulaer, MaartenHotopp, Julie C. DunningTodd, PeterHsieh, PeggyTognetti, SilviaHu, ZihuaTomida, JunyaHuang, Franklin W.Torres, JasonHunt, HarrietToth, KarolyHunter, JessicaTrono, DidierHyrien, OlivierTrumble, John T.Iguchi, TaisenTsuchihara, KtsuyaImamura, YukaTsuchimoto, DaisukeImaoka, TatsuhikoTsukahara, ToshifumiImatani, AkiraTurner, JillImig, JochenUlrich, HelleInácio, AngelaUsdin, KarenInagi, ReikoValdés, Ana ElisaIorizzo, MassimoValenzuela, ManuelIraqi, FuadValli, Adrian AlejandroIrato, PaolaVan Roon, Willeke M.C.Irish, ErinVan Roosbroeck, KatrienIshii, TetsuroVanDeHey, Justin A.Ishikawa, YuichiVanoirbeek, JeroenIshino, YoshizumiVargas-Alarcón, GilbertoIsles, Anthony R.Vasan, SenthilJacobs, HeinzVeening, Jan WillemJacobs, ThomasVeenstra, JanJamain, StephaneVerhoeven, Josine E.Jamilena, ManuelVerstraeten, AlineJanitz, MichaelVilla, AnnaJat, Parmjit S.Vingerhoets, GuyJeggo, PennyVisca, PaoloJelliffe, LauraVladimirov, VladimirJerónimo, CarmenVogel Ciernia, AnnieJetten, AntonVölker, JensJiang, Shu-YeVonk, Lucienne A.Jiang, XiVrabioiu, AlinaJiménez-Conde, JordiVyas, NoraJohnson, Eric TWachowiak, MattJohnston, Paul R.Walkley, CarlJoss-Moore, Lisa A.Wallace, DavidJournot, LaurentWalther, WolfgangJouve de la Barreda, Nicolás GuillermoWalworth, Nancy C.Jugo, Begoña M.Wan, LeiJurk, DianaWang, HongbingKagey, Jacob DWang, Jue D.Kaito, ChikaraWang, LizhongKale, AbhijitWang, XinjiangKandimalla, RajuWang, YongKang, Kwon-KyooWard, ChristopherKaplan, DanielWard, Judson A.Karanis, PanagiotisWatson, LaurenKetkar, AmitWeck, Karen E.Khanna, Kum KumWei, GanKhew-Goodall, YeesimWeisman, RonitKhobta, AndriyWeitzel, Joachim M.Kidane, DawitWhite, PeterKikawada, TakahiroWhyard, SteveKikuta, ShingoWilkins, Laetitia G.E.Kimura, TakahiroWilliams, Gwyn T.Klimentidis, YannWilson, David M.Klink, VincentWollman, AdamKloskowski, TomaszWolz, ChristianeKnoop, VolkerWong, JasonKnowles, Margaret A.Wong, Sek ManKoepp, Deanna MWu, Chih-HsiungKooy, FrankWu, Jiang I.Korolev, SergeyWurtele, HugoKorzhnev, DmitryWynant, NielsKosova, GulumWysocki, AnnetteKotterman, Melissa A.Xiao, XiaoKourti, AnnaXue, ChaoyangKraikivski, PavelYakovlev, Igor A.Kristensen, Lasse SommerYamagishi, HiroyukiKumar, RajivYamaguchi, KanjiKumari, DamanYan, ShanKundakovic, MarijaYan, YanKwon, Tae GyunYang, YonghuaLabate, Joanne A.Ye, Shui QingLacombe, BenoîtYeh, Jan-yingLajiness-O’Neill, RenéeYi, Joo-MiLamaze, Fabien C.Yildiz, AhmetLanubile, AlessandraYin, WenbingLefebvre, PhilippeYogev, OhadLeffak, MichaelYokoyama, KenichiLehmann, Alan R.Yoon, Sung-SooLei, BenfangYoung, Terry-LynnLeimkühler, SilkeYu, Yi-TaoLevanon, ErezZahn, Karl E.Levesque, CelineZandi, MichaelLewandoski, MarkZaritsky, AriehLi, FengzhiZebelo, Simon AtsbahaLi, GuojunZeng, LinLi, LinZhang, LixinLi, PingZhang, QingpengLi, QianfengZhang, YixiangLi, Xian-ZhiZhang, ZhiguoLian, Jane B.Zhao, LinlinLiaw, Shwu-JenZhou, HaiyanLiberi, GiordanoZhou, HuitongLigterink, WilcoZhou, QingxiangLinardic, Corinne M.Zhou, YunLing, HuiZhu, Guo-ZhangLingner, JoachimZhu, QianhaoLiu, FanZhu, YanminLiu, HongliangZuellig, RichardLiu, Huan-zhang


